# Time to Bloom

**DOI:** 10.1186/2041-9414-1-14

**Published:** 2010-11-04

**Authors:** Shweta Tikoo, Sagar Sengupta

**Affiliations:** 1 National Institute of Immunology, Aruna Asaf Ali Marg, New Delhi 110067, India

## Abstract

Bloom Syndrome (BS) is an autosomal recessive disorder due to mutation in Bloom helicase (referred in literature either as BLM helicase or BLM). Patients with BS are predisposed to almost all forms of cancer. BS patients are even today diagnosed in the clinics by hyper-recombination phenotype that is manifested by high rates of Sister Chromatid Exchange. The function of BLM as a helicase and its role during the regulation of homologous recombination (HR) is well characterized. However in the last few years the role of BLM as a DNA damage sensor has been revealed. For example, it has been demonstrated that BLM can stimulate the ATPase and chromatin remodeling activities of RAD54 *in vitro*. This indicates that BLM may increase the accessibility of the sensor proteins that recognize the lesion. Over the years evidence has accumulated that BLM is one of the earliest proteins that accumulates at the site of the lesion. Finally BLM also acts like a "molecular node" by integrating the upstream signals and acting as a bridge between the transducer and effector proteins (which again includes BLM itself), which in turn repair the DNA damage. Hence BLM seems to be a protein involved in multiple functions - all of which may together contribute to its reported role as a "caretaker tumor suppressor". In this review the recent literature documenting the upstream BLM functions has been elucidated and future directions indicated.

## Role of protein phosphorylation in response to DNA damage

Signal transduction during DNA damage response is mediated by two proximal sensory kinases, ATM (ataxia telangiectasia-mutated) and ATR (*ATM*-Rad3-related) [1,2]. ATM and ATR initiate the signaling cascade via phosphorylation of its downstream checkpoint effector kinases, Chk1 and Chk2 [3]. ATR and Chk1 predominantly sense the damage in response to the stalling and subsequent collapse of the replication forks (called stalled replication forks), leading to replication arrest. On other hand ATM and Chk2 are involved in response to double strand breaks (DSBs), typically generated *in vivo *by exposure of cells to ionizing radiation (IR) or drugs like neocarzinostatin or bleomycin. Both stalled replication forks and DSBs lead to the generation of nuclear chromatinized foci called stalled replication foci and ionizing radiation-induced foci (IRIF), respectively. Replication arrest can also lead to the generation of DSBs [4], thereby hinting at partial common mechanistic framework in response to two common forms of DNA damage. ATM/ATR along with Chk1/Chk2, which accumulate at the chromatinized structures, are known to phosphorylate extensive network of downstream substrates in response to DNA damage [5].

**Figure 1:**
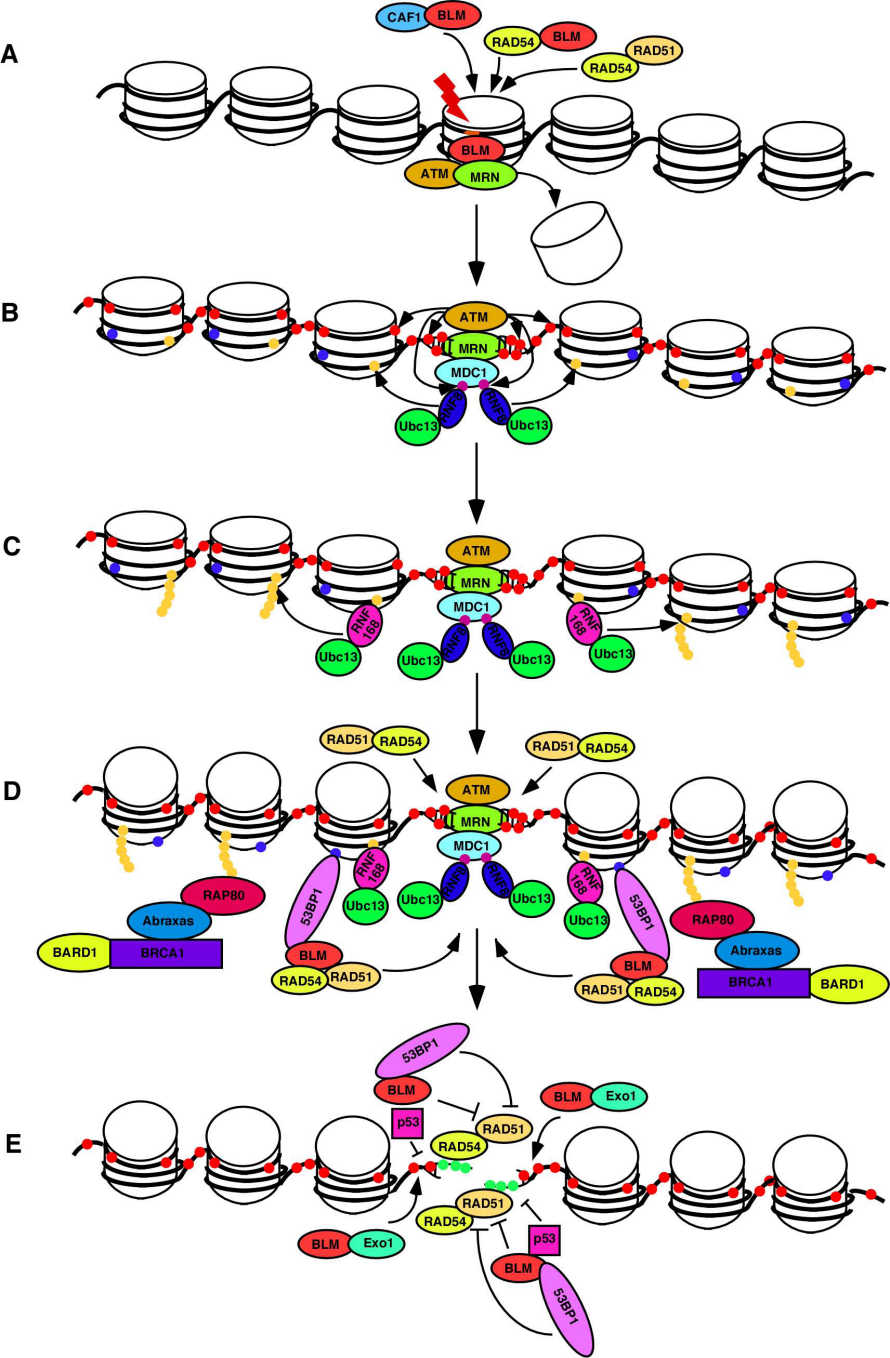
**Proposed model for the functions of BLM helicase during DNA damage response**. (A) DSBs (red line) are recognized after BLM and/or RAD51-stimulated RAD54-dependent chromatin remodeling. BLM affects chromatin organization by interacting with and regulating the function of CAF-1. On remodeled chromatin, BLM accumulates and helps in the optimal ATM activation and MRN complex accumulation. (B) MRN complex promotes H2AX phosphorylation (γH2AX, red dots) which recruits MDC1. MRN complex, stabilized on the DNA lesion by MDC1, promotes further accumulation of activated ATM. ATM phosphorylates MDC1 (purple dots), promoting the binding and recruitment of RNF8/Ubc13 complex, which catalyzes the Lys63-linked ubiquitylation of H2A and H2AX (yellow dots), causing a more accessible conformation of the chromatin. (C) RNF8/Ubc13 ubiquitylated histones recruits of RNF168. RNF168/Ubc13 attaches K63 linked polyubiquitin moieties to RNF8-ubiquitylated histones (yellow dots). (D) Poly-ubiquitylated histones recruits RAP80, which helps in the accumulation of Abraxas/BRCA1/BARD1 at DSBs. Constitutive methylation of histones H3 and H4 (blue dots) are probably exposed due to RNF168/Ubc13-dependent ubiquitylation. This results in the efficient recruitment of 53BP1 to the site of DNA damage. BLM again accumulates on the lesion in a 53BP1-dependent manner. Pro-recombinogenic proteins RAD51 and RAD54, interact with BLM, and accumulate at DSBs. (E) BLM functionally interacts with its partners like RAD51, RAD54, 53BP1 and p53 during HR. RAD51 binds to the single stranded DNA by displacing replication protein A (green dots). While BLM, 53BP1 and p53 have anti-recombinogenic property; BLM also has a pro-recombinogenic resection function in coordination with Exo1.

The protein that was initially demonstrated to accumulate at the site of IRIF was the phosphorylated form of histone variant H2AX (γH2AX) [6] (Figure 1B). However subsequently it was observed that H2AX phosphorylation was dispensable for the initial recognition of DNA breaks and was instead proposed to concentrate proteins in the vicinity of DNA lesions [7]. Since then a growing number of proteins, containing either or both the phospho-protein binding motifs BRCA1 C-terminal (BRCT) and forkhead associated (FHA) domains, have been identified to be present both at IRIF and sites of stalled replication.

One such FHA-BRCT domain containing protein that accumulates at the sites of DNA damage is the mediator of DNA damage checkpoint 1 (MDC1) (Figure 1B). Recruitment of MDC1 occurs in a BRCT-dependent manner by binding to the C-terminal phosphorylated tail of H2AX [8]. MDC1 is required for intra-S phase DNA damage checkpoint [9-11]. At the IRIF, MDC1 acts like a molecular adaptor required for the localization of a number of other DNA damage response proteins including MRE11-RAD50-NBS1 (MRN) complex.

NBS1 (named for Nijmegen breakage syndrome; also called nibrin), a key member of the MRN complex, accumulates at the IRIF due to its own phospho-peptide binding FHA-BRCT domain [12] (Figure 1B). MDC1 stabilizes NBS1 at the sites of DNA damage, thereby promoting further accumulation of the MRN complex and activated ATM [13,14]. Recent studies have revealed that phosphorylation of Ser-Asp-Thr-Asp (SDTD) repeats within MDC1 mediate its interaction with the FHA-BRCT domain of NBS1. This phospho-dependent interaction mediates the retention of MRN complex at the sites of DNA damage, thereby ensuring optimal S-phase checkpoint activation [15-18].

## Role of protein ubiquitylation in response to DNA damage

The localization of conjugated ubiquitin at the sites of DNA damage had been demonstrated quite sometime back [19,20]. Subsequently receptor associated protein 80 (RAP80) that contains tandem ubiquitin interacting motif (UIM) and binds to Lys63 and Lys6 polyubiquitin chains was identified at the IRIF [21,22]. RAP80 targets Breast Cancer gene 1 (BRCA1) and BRCA1-associated Ring Domain 1 (BARD1) complex to the sites of DNA damage utilizing its association with Abraxas (ABRA1) [23-25]. The recruitment of BRCA1/BARD1 complex is required for its function in DNA damage resistance, intra-S and G2/M checkpoint control as well as DNA repair [25].

Interestingly around this time it was reported that the E3 ligase, Ring finger protein 8 (RNF8) assembles at the site of DSBs via the interaction of its FHA domain with the conserved Thr-Gln-any amino acid-Phe (TQXF) motif in MDC1. Phosphorylation of the MDC1 TQXF motifs by ATM and ATR is required for the interaction with RNF8 [26-28] (Figure 1B). Once recruited RNF8 and E2 conjugating enzyme Ubc13 catalyzes the Lys63-linked ubiquitylation of H2A and H2AX. This ubiquitylation promotes the transition of chromatin into a more accessible conformation leading to the recruitment of p53 binding protein 1 (53BP1) and RAP80/Abraxas/BRCA1 complex to the DNA damage foci [26-29].

A subsequent study reported that a patient with RIDDLE syndrome (radiosensitivity, immunodeficiency, dysmorphic features and learning difficulties) was defective in the recruitment of 53BP1 and BRCA1 to the DSBs [30], indicating the presence of another protein in the RNF8-dependent 53BP1 recruitment process. A siRNA screen using 53BP1 foci formation as the readout revealed an E3 ligase, Ring finger protein 168 (RNF168), as the gene mutated in RIDDLE syndrome [31,32]. RNF168, which contains two motifs that interact with ubiquitin (MIU), is recruited to the to sites of DNA damage by binding to ubiquitinated H2A. The assembly of RNF168 at DSBs occurs in RNF8-dependent manner and leads to the amplification of RNF8-dependent substrate ubiquitylation (Figure 1C). RNF168/Ubc13 mediated histone poly-ubiquitylation recruits RAP80 to the sites of DNA damage, which in turn helps in the accumulation of Abraxas/BRCA1/BARD1 at DSBs (Figure 1D).

53BP1 accumulates at IRIF by interacting with methylated histones H3 and H4 via its Tudor domain [33,34]. Histones H3 and H4 are constitutively methylated. However exposure to DNA damage causes a transition of histone H3 and H4 to a more accessible conformation due to RNF8/RNF168/Ubc13-dependent poly-ubiquitylation, which exposes the H4-K20 and/or K3-K79, methylated histones. This results in the recruitment of 53BP1 to the site of DNA damage (Figure 1D). Hence lack of both RNF8 and RNF168 leads to disruption in the recruitment of 53BP1.

## Bloom (BLM) helicase and DNA damage response

### BLM helicase and cancer

Bloom Syndrome (BS) is an autosomal recessive disorder that is associated with predisposition to cancer [35]. BS is characterized by proportional dwarfism, sun-induced chronic erythema, type II diabetes, male infertility and female subfertility and frequent infections due to immune deficiency. The BS afflicted individuals are predisposed to cancers. However unlike other cancer predisposition syndromes, BS patients suffer from almost all the major types of cancer [36]. This indicates that BLM is possibly involved at an early stage during neoplastic transformation - a step that maybe common for all forms of cancer. Hence understanding the cascades which regulates BLM functions and also deciphering the processes that the helicase itself regulates can give clues regarding the "common master regulatory step" which may precede the divergent epigenetic and genetic alterations that subsequently drive tumor formation.

The mean age of cancer diagnosis in BLM patients is 24 years and death is generally associated before the age of 30 [36,37]. Germ line mutations in *BLM *give rise to BS. Though BS patients are rare, BLM heterozygotes that carry a *BLM *mutation may be faced with a higher probability of developing colorectal cancer [38]. The exact percentage of BLM heterozygous individuals in general population is unknown. However in Ashkenazi Jewish population the frequency of BS is approximately 1 in 48,000. This is due to a founder effect, approximately 1% of the Ashkenazi Jewish population being heterozygous carriers of the BLM^*Ash *^mutation (a six nucleotide deletion and a seven nucleotide insertion at position 2281 of the cDNA) [39]. Transgenic mouse model studies also support the hypothesis that carriers of a single defective *BLM *allele are cancer prone [40]. Based on recent studies (described below) is quite possible that BLM is involved in the detection, transmission and finally the resolution of damaged DNA - in collaboration of other stage specific regulatory partners. Hence it can be hypothesized that the lack of BLM, may lead to a change in the stoichiometry of the proteins involved in DNA damage sensing and repair, which in turn may have an adverse effect during the neoplastic transformation process.

### Multiple functions of BLM helicase during DNA damage response

Though the functions of BLM in the resolution of DNA damage are well characterized, the role of this helicase in the DNA damage response is yet to be fully deciphered. The response of BLM to DNA damage signal is a direct consequence of the changes in the intranuclear localization of the helicase. In asynchronous cells BLM is found to be in promyelocytic leukemia nuclear bodies (PML NBs) and nucleolus [41,42]. Exposure to replication inhibitors (like hydroxyurea, HU) results in relocalization of BLM to the sites of stalled replication forks. It has been recently demonstrated that Chk1 constitutively phosphorylates BLM at Ser646, and this specific phosphorylation event rapidly decreases after exposure to DNA damage [43]. Lack of Ser646 phosphorylation post-DNA damage results in diminished interaction of BLM with nucleolin and PML isoforms and consequently leads to decrease in the accumulation of the helicase in nucleolus and PML NBs. Instead post-damage BLM colocalizes and associates with MRE11-Rad50-NBS1 complex [43-45], ATM [46,47] and ATR [45,48]. Both ATM and ATR phosphorylate BLM, indicating a possible role of the helicase in the recognition of DSBs and stalled replication [47,48].

Multiple lines of evidence exist indicating that BLM may function very early in response to DNA damage (Figure 1A). BLM is induced by treatment of cells with γ-irradiation in an ATM independent manner. This induction depends on G2 delay because it fails to occur when G2 phase is prevented or bypassed [49]. Secondly, ATR and ATM-dependent intranuclear trafficking of BLM helicase also occurs during replication stress, which ensures optimal ATM activation and 53BP1 focus formation [50]. Hence cells from BS patients undergo delayed assembly of BRCA1 and NBS1 repair complexes at stalled replication forks [44]. Thirdly, both endogenous and overexpressed BLM accumulates at sites of laser-induced DSBs within 10 seconds and colocalizes with γ-H2AX and ATM. The early accumulation of BLM at DSBs is independent of ATM, RAD17 and NBS1 [51]. Finally, absence of BLM impairs the ability of Chromatin Accessibility Factor-1 (CAF-1) to be mobilized at the sites of DNA damage within the nucleus [52], thereby indicating that BLM may have an effect at the chromatin remodeling stage. Incidentally apart from its functions during the presynaptic, synaptic and post-synaptic phases of HR [53,54], RAD54 also functions as a chromatin remodeller, both *in vitro *[55-57] and *in vivo *[58]. BLM stimulates the ATPase and chromatin remodeling activities of RAD54 *in vitro *[59], and is therefore potentially capable of enhancing the accessibility of the DNA damage sensor proteins to the DNA lesion *in vivo*. Using Fluorescence Recovery After Photobleaching (FRAP) the residence time of BLM in the HU-induced foci is only 7.2 seconds, providing evidence about the transitory nature of BLM during the sensing and recognition of DNA damage.

However it has been reported that BLM also accumulates at the site of stalled replication around 1 hour post-HU-treatment [60,61]. This second wave of BLM accumulation depends on 53BP1 with which it physically interacts (Figure 1D). The accumulation of BLM/53BP1 foci and the physical interaction between them was dependent on phosphorylation-mediated interactions [62]. These observations have led to the hypothesis that BLM also plays a role at a relatively later stage in the hierarchy of proteins accumulating at the site of damage. According to this model BLM acts as a "molecular node" in response to replication stress during S-phase checkpoint. During this step BLM may integrate the signal(s) obtained from the upstream damage recognizing proteins and coordinate with repair and recombination proteins downstream to efficiently remove the deleterious lesion [60,61].

Hence BLM seems to have roles in multiple phases of the DNA damage response pathway. In the immediate early phase BLM acts independently in the pathway, not even requiring other known early sensors of DNA damage like γH2AX, MDC1 and 53BP1. In the intermediate phase BLM acts as a molecular node, physically and functionally interacting with multiple proteins already associated at the site of damage and acting either in hierarchical or combinatorial manner so that the downstream repair proteins can receive and process the DNA damage signal. It is in this last step, the repair phase (Figure 1E), BLM acts in combination with its partners like RAD51 [60,63-67], RAD54 [59,68], 53BP1 [62,66] and p53 [60] to remove the deleterious lesions so that the genome integrity can be maintained. While the predominant function of BLM is anti-recombinogenic [59], the helicase also interacts with human exonuclease 1 (Exo1) to resect DNA and initiate the process of DNA repair [69,70]. This process indicates a pro-recombinogenic function of BLM. Detailed description of BLM functions in the repair phase can be obtained from several excellent recent reviews [71-75], and hence have not been described in detail here.

## Conclusions

BLM helicase has attracted much attention due to the hyper-recombinogenic phenotype of BS patients and their predisposition to almost all forms of cancers known to human. Since BLM was first discovered to be a helicase [76], its *in vitro *biochemical role during HR had become the focus of intense research. Maybe due to the above reason and also possibly due to the non-availability of the desired tools and reagents, initial studies deciphering the *in vivo *cellular functions of BLM had been much fewer and less well characterized. However in the last half decade using a range of immortalized genetically engineered cell lines, specific antibodies that recognize endogenous BLM or its phosphorylated forms and with the widespread availability of robust microscopic techniques, the focus has shifted to towards deciphering how the helicase functions in a cellular context. This review had aimed to summarize all the important findings in this emerging field.

Perhaps the most important message that emerges is that BLM helicase has a fascinating "double life" apart from its well-characterized role as a helicase functioning during HR. Hence the functions of BLM are not confined to its role during HR but much earlier when the cells are exposed to the deleterious lesions. Hence BLM acts as a sensor, transmitter and finally the effector at different steps during the entire DNA damage signaling cascade - effectively acting as the "caretaker tumor suppressor" [36]. The fact that BS patients are predisposed to almost all forms of cancers can be interpreted to indicate that certain functions common to neoplastic transformation process is being regulated by BLM. Apart from HR, other repair pathways are also employed by eukaryotic cells [77,78], on which BLM does not seem to exert much or any control. Hence regulation of repair pathways by BLM (essentially HR) may not be the only reason for the wide spectrum cancer phenotype observed in BS patience. It can be argued that the "caretaker tumor suppressor" function of BLM at least partially depends on its upstream DNA damage sensor and transmission functions.

It is perhaps important to point out the similarity of the proposed multiple functions of BLM with those of NBS1 and 53BP1. One of the first functions assigned to MRE11-RAD50-NBS1 (MRN) complex was a role in the repair of DNA double strand breaks [79]. Initially NBS1 was shown to be a substrate for ATM phosphorylation. Thus cells from NBS patients were defective in checkpoint response [79,80]. However evidence is accumulating that MRN complex also functions upstream of ATM [81,82]. Recent mouse models have confirmed that NBS1 is required for activation of ATM during DNA double strand breaks [83]. Similarly 53BP1 has been well characterized as a DNA damage sensor protein recruited to the site of damage by an ubiquitylation-dependent cascade [84,85]. However recently a role of 53BP1 in DNA repair has been established. 53BP1 has an anti-recombinogenic function [86], which is dependent on both BLM [66] and BRCA1 [87]. 53BP1 also affects the classical and alternate end-joining pathway during class switch recombination [88,89]. Hence it is quite possible that proteins involved in either sensing and transmission of DNA damage signal and those regulating DNA repair processes can have additional functions which are different than that presently ascribed to them.

Finally the question arises - what are the future directions of research on BLM especially in relation to its role as a damage sensor. It remains to be firmly established whether BLM can actually act as the universal damage sensor and transmitter as most of the studies until now have been done on cells containing either stalled forks or DSBs. The study of BLM post-translational modifications (PTMs) individually and in combination, and how the PTMs affect BLM functions during signal recognition and transmission is bound to be of much interest to researchers. But perhaps in the genomics era, the time has come not to look at BLM functions in isolation. With the help of high throughput technologies it is imperative that BLM functions are analyzed on a "global" scale perhaps in conjunctions with the dynamicity of its interaction with its chromatin (which is its actual *in vivo *substrate) but also with its stage specific protein partners, a few known but perhaps many unknown. The information available till date already indicates that the time has come for BLM helicase to bloom and show its true potential to the researchers.

## Competing interests

The authors declare that they have no competing interests.

## Authors' contributions

Both ST and SS have been involved in conceptualizing and writing the review. Both the authors read and approved the final version of the manuscript.

## Authors' information

ST received her Masters in Biotechnology from University of Jammu in 2007. She is a graduate student in SS's lab and her interests include the study of post-translational modifications in proteins involved in DNA damage response.

SS received his Ph.D. in 1997 from the Indian Institute of Science, India, for his research on the regulation of nitrogen assimilation in yeasts. During his postdoctoral studies at the Institut de Génétique et de Biologie Moléculaire et Cellulaire (IGBMC), France, he examined the functional interaction between the tumor suppressor p53 and the glucocorticoid receptor. Subsequently, in the Laboratory of Human Carcinogenesis, National Cancer Institute, Bethesda, USA, SS studied the inter-regulatory roles of p53 and RecQ helicases. His ongoing research interests involve the study of the interactions between the members of the eukaryotic signal-transduction cascade and the DNA-repair and -recombination machinery. He joined the National Institute of Immunology, India, as a group leader in September 2004.
